# Longitudinal Analysis of Intracochlear Electrocochleographic Amplitude Patterns in Cochlear Implant Recipients

**DOI:** 10.1097/AUD.0000000000001793

**Published:** 2026-02-06

**Authors:** Marlies Geys, Ahmet Kunut, Rahel Bertschinger, Ivo Dobrev, Andrea Kegel, Christof Röösli, Alexander Huber, Adrian Dalbert, Flurin Pfiffner, Leanne Sijgers

**Affiliations:** 1 Department of Otorhinolaryngology, Head & Neck Surgery, University Hospital Zurich, University of Zurich, Zurich, Switzerland.

**Keywords:** Cochlear health, Cochlear implant, Computed tomography, Electrophysiology, Electrocochleography, Hearing preservation

## Abstract

**Objectives:**

Intracochlear electrocochleography (ECochG) in cochlear implant (CI) recipients is a potential tool for monitoring cochlear function during and after electrode array (EA) insertion. However, mechanisms underlying ECochG amplitude variations along the cochlear duct, and their significance for hearing preservation (HP), remain unclear. Therefore, a longitudinal study was conducted to monitor maximum ECochG amplitude and its tonotopic location from EA insertion to 1 yr postimplantation. It was hypothesized that changes in maximum amplitude (>30%) and/or shifts in its location (>1 octave) across timepoints reflect intracochlear alterations associated with residual hearing changes.

**Design:**

ECochG recordings were obtained in 80 adult CI recipients with measurable residual hearing. For Contour Advance (CI612) and Slim Straight (CI622) arrays (Cochlear Ltd.), recordings were taken from every second intracochlear electrode. For HiFocus SlimJ and MidScala arrays (Advanced Bionics LLC), recordings were obtained from all electrodes. Measurements were conducted at four timepoints: (1) intraoperatively, during EA insertion (Intraop1), (2) intraoperatively, immediately after full insertion (Intraop2), (3) approximately 7 wk after surgery (Postop1), and (4) approximately 1 yr after surgery (Postop2). 500 Hz tone bursts were used for acoustic stimulation and the magnitude of the difference between responses to alternating-polarity stimuli was analyzed. Tonotopic electrode locations were determined from postoperative cone beam computed tomography scans. Pure-tone audiograms were obtained preoperatively and at approximately 7 wk and 1 yr postoperatively. HP was determined using the HEARRING group formula.

**Results:**

Maximum ECochG amplitudes remained largely stable intraoperatively, with no significant difference between Intraop1 and Intraop2 in complete-case analysis (n = 44). In contrast, a significant decrease in maximum amplitude was observed between Intraop2 and Postop1 (*p* < 0.001). Participants with >30% amplitude reduction between the 2 intraoperative recordings (Intraop1 versus Intraop2) did not differ significantly in HP from those with stable amplitudes. However, those showing a >30% reduction in the early postoperative period (Intraop2 versus Postop1) showed significantly lower HP (*p* = 0.028). Nonapical peak location during Intraop1 occurred in 41% of the cases, although tonotopic location of the maximum peak during insertion monitoring (Intraop1) did not show a relationship with HP. Tonotopic location shifts of the maximum amplitude (>1 octave) were observed in a small subset of cases between consecutive recordings up to Postop2. However, peak location changes (apical, basal, stable) were not associated with significant differences in HP.

**Conclusions:**

Our results suggest that nonapical peak patterns are not necessarily markers of insertion trauma and may instead reflect variability in cochlear integrity (e.g., dead regions). Peak location during insertion monitoring was not associated with postoperative HP, and both maximum amplitude and tonotopic peak location remained stable intraoperatively. In contrast, early postoperative reductions in ECochG amplitude were common and associated with HP, highlighting the need to investigate strategies to minimize early intracochlear reactions. Overall, the study demonstrates the value of ECochG for monitoring intracochlear processes over time.

## INTRODUCTION

A cochlear implant (CI) is an implantable hearing device that restores auditory perception in individuals with severe to profound hearing loss by electrically stimulating the auditory nerve fibers through intracochlear electrode contacts. Increasingly, CI recipients have residual hearing before implantation, which, when preserved postsurgery, offers additional benefits, such as improved music perception and enhanced speech understanding (Dalbert et al. 2016a; Bester et al. 2017; Schaefer et al. 2021). However, preserving residual hearing remains challenging. Delicate cochlear structures, including the basilar membrane (BM) and hair cells, can be affected during electrode array (EA) insertion. Furthermore, postinsertion intracochlear processes, such as inflammation or intracochlear tissue growth (fibrosis or ossification), can alter cochlear mechanics and affect acoustic hearing (Choi & Oghalai 2005; O’Leary et al. 2013; Geerardyn et al. 2023).

Electrocochleography (ECochG), an electrophysiological method used to derive early, acoustically evoked potentials, may serve both as an intraoperative monitoring tool during CI electrode insertion to minimize insertion trauma (Dalbert et al. 2020; Bester et al. 2022; Harris et al. 2022; Sijgers et al. 2023) and as a biomarker of cochlear function (Abbas et al. 2017; Bester et al. 2017; Soulby et al. 2021; Tejani et al. 2023; Walia et al. 2023). Recorded ECochG signals consist of different response generators, including hair cell and neural generators. The cochlear microphonic is the most commonly used ECochG component, as its large amplitude enables detection of magnitude changes with relatively few stimulus repetitions (Calloway et al. 2014) and can be recorded in most CI users, including some with only minimal residual hearing (Harris et al. 2017b; Dalbert et al. 2018). A common method to extract the latter is subtracting two ECochG recordings with alternating polarity, resulting in a “difference curve” (DIFF) which predominantly reflects the hair cell activity. However, interference between the hair cell and neural signals cannot be entirely excluded (Forgues et al. 2014). ECochG measurements can be obtained from a recording location close to the cochlea, such as on the promontory (extracochlear ECochG, e.g., Dalbert et al. 2016b), intracochlearly through the electrode contacts on the EA (Campbell et al. 2016), or simultaneously (Sijgers et al. 2021). The main advantages of intracochlear over extracochlear ECochG recording sites are larger response amplitudes, thanks to the proximity to the generators, resulting in improved signal to noise ratio (Dalbert et al. 2015), and the ability to record from different intracochlear electrodes along the cochlear duct, enabling enhanced tonotopic resolution, even postoperatively.

Low-frequency acoustic stimulation at high intensity is typically used to evoke ECochG potentials, as residual hearing in CI recipients is often preserved in the apical region of the cochlea, where low-frequency resonance occurs. As a result, in the majority of the CI users, the largest ECochG responses are generated closest to the apex, which is approached toward the end of the insertion of the EA (Harris et al. 2017a). Despite this general trend, different ECochG response patterns have been described in the literature, showing decreases or steady responses of the ECochG magnitude from the beginning to the end of EA insertion, maximum amplitude at mid-insertion, or a series of smaller amplitude fluctuations during insertion, either with or without considering phase changes (Harris et al. 2017a, 2024; Koka et al. 2018; Giardina et al. 2019; Ramos-Macias et al. 2019; Buechner et al. 2022; Haumann et al. 2024b; Dalbert et al. 2025). Furthermore, postinsertion measurements have identified distinct response patterns along the EA, characterized by apical, mid, basal, or flat peaks, with the maximum amplitude occurring in the apical, middle, or basal cochlear regions, or in some cases, no discernible peak (Bester et al. 2017, 2023; Schuerch et al. 2023). Even shifts in peak patterns have been reported, with a study demonstrating that a tonotopic shift toward a more basal location at 3 mo postoperatively, relative to the position immediately postinsertion, is associated with greater hearing loss at the 3-mo time point (Bester et al. 2023).

Different mechanisms can contribute to the distinct ECochG response patterns observed. Trauma-related factors such as EA insertion trauma, whether or not accompanied by dislocation of the EA from the scala tympani into the scala vestibuli, can reduce ECochG magnitude in the affected region (Dalbert et al. 2016b, 2019; Koka et al. 2018; Riggs et al. 2019; Lenarz et al. 2022; O’Leary et al. 2023; Harris et al. 2024). Mechanical influences can arise from contact between the EA and the BM or from postoperative intracochlear tissue growth, both of which can reduce BM displacement and disrupt the acoustic traveling wave (Choi & Oghalai 2005; Kiefer et al. 2006; Quesnel et al. 2016; Bester et al. 2023). Cochlear health variability, such as the presence of dead regions along the BM, related to etiology or degree of hearing loss, can affect ECochG responses (Bester et al. 2017; Giardina et al. 2019; van Gendt et al. 2020; Andonie et al. 2025; Dalbert et al. 2025). At last, measurement-related factors such as high-intensity stimuli used to evoke potentials can introduce interactions between responses with different phases generated in different cochlear regions, causing constructive and destructive interactions in the recorded signal (Giardina et al. 2019; Hutson et al. 2021; Sijgers et al. 2021; Suntinger et al. 2022), and level-dependent deviations from the Greenwood map, with ECochG peaks shifting by up to an octave at high levels (Sagi & Svirsky 2025; Walia et al. 2025).

The implications of the various ECochG patterns along the cochlear duct remain controversial. A longitudinal study monitoring participants from CI electrode insertion to 1 yr postimplantation at various tonotopic locations could enhance the understanding of mechanisms responsible for these patterns. In addition, investigating ECochG amplitude patterns across CI recipients implanted with different EAs requires precise consideration of tonotopic recording locations within the cochlea, an aspect often overlooked in ECochG research. Factors such as EA type and length, surgical insertion approaches, insertion depth, and individual anatomical variations in cochlear size all influence the location of the recording electrodes and must be considered when analyzing ECochG responses along the EA (Geys et al. 2024; Andonie et al. 2025). Therefore, the key objective of this study is to provide new insights into ECochG amplitude patterns, with respect to the individual tonotopical locations of the recording electrodes, in a longitudinal observational study. The first aim is to compare intracochlear ECochG amplitude patterns, acquired continuously during CI insertion, after full array insertion, and at two timepoints postoperatively within the first year, by monitoring both the maximum ECochG amplitude and its corresponding tonotopic location. The second aim is to investigate the relationship between changes in ECochG amplitude patterns over time and hearing preservation (HP) outcomes. It was hypothesized that a reduction in ECochG amplitude and/or a shift in the tonotopic location of the maximum amplitude between recordings would be associated with a greater degree of postoperative hearing loss. These measurable changes are expected to reflect underlying intracochlear trauma or physiological alterations affecting residual cochlear function.

## MATERIALS AND METHODS

### Participants

Eighty adult participants were included in this prospective, observational, longitudinal, single-center study. Eligibility criteria required participants scheduled for CI surgery with some degree of residual low-frequency acoustic hearing, defined as 500 Hz thresholds of ≤115 dB HL (maximum output of the audiometer). A broad range of preoperative residual hearing was included, as previous studies have shown that meaningful ECochG responses can be obtained even in some CI candidates with minimal residual hearing before surgery (Harris et al. 2017b; Dalbert et al. 2018). Participants with confirmed inner ear or auditory nerve malformations were excluded. The study was approved by the local ethics committee and conducted in accordance with the Helsinki Declaration. All participants provided written informed consent before surgery.

### ECochG Recording Procedure and Equipment

Intracochlear ECochG responses were recorded at four timepoints: (1) intraoperatively during EA insertion using the CI’s most apical electrode (Intraop1), (2) intraoperatively, immediately after full insertion of the EA from multiple electrodes (Intraop2), (3) approximately 7 wk after surgery, again using multiple CI electrode contacts (Postop1), and (4) approximately 1 yr after surgery (Postop2). Intraop2, Postop1, and Postop2 are referred to as sweep recordings, obtained from 11 recording electrodes (every second electrode, 2-22) with Cochlear Ltd. (Sydney, Australia) arrays, or all 16 electrodes with Advanced Bionics LLC (Valencia, CA, USA) arrays. Average inter-recording EL distances were 1.8 mm for Slim Straight CI622 (Cochlear Ltd.), 1.2 mm for Contour Advance CI612 (Cochlear Ltd.), 1.3 mm for HiFocus SlimJ (Advanced Bionics LLC), and 0.975 mm for HiFocus MidScala (Advanced Bionics LLC). Postop2 was performed only if valid ECochG responses were present during Postop1. This approach was based on the assumption that absent responses at an earlier timepoint are unlikely to reappear in later measurements. Consequently, unnecessary testing was avoided in participants without expected measurable responses. The number of participants excluded at each stage is reported transparently. Intraoperatively, once the apical electrode of the CI EA was placed into the cochlear fluid via the round window (RW) incision or cochleostomy opening, electrode conditioning and impedance recording were performed. During insertion of the EA into the scala tympani, the surgeon indicated each electrode contact as it passed through the cochlear entrance. These timestamps were marked in the ECochG software, up to the final timestamp at full insertion. After fixating the EA, electrode conditioning and impedance measurements were performed for all recording electrodes. Subsequently, the electrode sweep measurement (Intraop2) was initiated. This measurement was repeated after approximately 7 wk (Postop1) and after approximately 1 yr postoperatively (Postop2). Any contralateral hearing device was removed during ECochG recordings.

Recordings were performed with the Cochlear Research Platform (CRP, v1.2) from Cochlear Ltd. or the Active Insertion Monitoring System (AIM, OM Suite v1.1) from Advanced Bionics LLC. Additional details on the system setup are described in Schuerch et al. (2022). A calibrated probe microphone (ER-7C; Etymotic, Inc., Elk Grove Village, IL, USA) was placed in the external auditory canal to evaluate the acoustic stimulation. 500 Hz tone bursts of 13 msec (CRP) and 50 msec (AIM) with alternating starting phases were used for acoustic stimulation with a rise-and-fall time of 1 msec (CRP) and 5 msec (AIM). During insertion monitoring (Intraop1) with the CRP, a block of four alternating tone bursts was presented with a 1 Hz rate until full insertion. The AIM system used continuous stimulation at a 14 Hz repetition rate during insertion. For sweep measurements, 400 repetitions (200 rarefactions and 200 condensations) were recorded for each recording electrode with the CRP, while with AIM, either 200 repetitions (100 rarefactions and 100 condensations) were recorded, or the recording was terminated early on reaching a signal to noise ratio of 24 dB, determined by comparing energy in stimulus-frequency bins to adjacent bins. The recording window was set at 19 msec (CRP) and 50 msec (AIM). A suprathreshold level of the acoustic stimulation was chosen based on the preoperative audiogram to avoid any intolerance of the stimulation level when repeating the measurement postoperatively. The intensity ranged from 93 to 115 dB HL. The peak-to-peak amplitude of the acoustic stimulation, monitored using the in-situ probe microphone, was saved intraoperatively using an oscilloscope (Rohde & Schwarz, RTB2004) and matched in postoperative tests. Due to postoperative comfort limitations, minor adjustments were sometimes necessary. Recordings with stimulus-level deviations >5 dB SPL were excluded from amplitude comparisons but retained for peak-location analyses, as small variations are not expected to alter the location of the maximal ECochG response.

### Audiogram

Pure-tone audiograms were conducted within 6 mo before surgery, approximately 7 wk after surgery, and approximately 1 yr after surgery. Audiograms were performed in accordance with ISO 8253-1:2010 (ISO 2010). Air conduction threshold values were measured at 0.125, 0.25, 0.5, 0.75, 1, 1.5, 2, 3, 4, 6, and 8 kHz. Bone conduction thresholds were obtained for 0.25, 0.5, 1, 2, and 4 kHz. Contralateral masking was implemented when needed. Missing interoctave frequencies were interpolated, and the pure-tone average (PTA) was calculated from all frequencies. The maximum output of the audiometer with the air conduction headphones was 70 dB HL for 0.125 kHz, 90 dB HL for 0.25 and 8 kHz, 110 dB HL for 6 kHz, and 115 dB HL for all other frequencies. Four HP categories were defined by using the HEARRING group classification (Skarzynski et al. 2013). A >75% preservation was classified as “Complete HP,” >25 to 75% as “Partial HP,” 0 to 25% as “minimal HP,” and no measurable hearing as “Loss of hearing.” HP at the first postoperative measurement is compared with the preoperative audiogram (HP_1_). In addition, the low-frequency PTA (LFPTA) was calculated as the average of 0.25, 0.5, and 1 kHz, and both the mean LF reduction and the overall PTA reduction were reported.

### Tonotopic Location of the Recording Electrodes

Clinical cone beam computed tomography (CT) was performed postoperatively, typically up to 3 mo post-surgery, with a slice thickness of 0.15 mm. These scans were analyzed using OTOPLAN (v3.0.0) (CASCINATION AG, Bern, Switzerland) to extract the cochlear duct length at the level of the organ of Corti/BM (CDL_OC_). The cochlear view was manually defined by two investigators (M.G. and R.B.), who also marked the points to measure the diameter, width, and height of the cochlea, resulting in an automatically calculated CDL_OC_. The mean CDL_OC_ of the two investigators was used afterward. In addition, any scalar dislocation of the EA was examined by the two investigators in the postoperative CT scan and confirmed by an experienced otorhinolaryngologist (A.D.). The exact tonotopic locations of the recording electrodes were then determined from the CT scans using a MATLAB (R2022b) script, freely accessible for generating the ZH-ECochG Bode Plot (Geys et al. 2024). The script was updated to better account for different insertion approaches (RW and cochleostomy) and various EA types, which result in different positions relative to the OC/BM. The adapted version of the script includes an additional manual selection of the cochlea’s outer wall and a projection of the apical and basal electrodes to the sextic polynomial at the outer wall in polar coordinates. The change to the polar coordinate system was included to simplify the estimation of the spiral configuration of the cochlea. Afterward, the insertion depth of the electrodes (mm) is calculated by interpolating between the most apical and basal electrode and converted using a 0.9 ratio to the level of the OC (Dhanasingh 2019), where the ECochG’s cochlear microphonic potentials are generated. The respective tonotopic frequencies are then calculated using the Greenwood Function based on the individual CDL_OC_, consistent with the original version of the script The updated version of the script can be found on https://github.com/OtoBM/ZH-ECochG-Bode-Plot. If no postoperative imaging was available or image quality was insufficient to determine the electrode’s location, the average tonotopic frequencies from participants with the same EA were used.

### Data Preparation

ECochG recordings were analyzed in MATLAB (R2022b) (MathWorks, Inc., Natick, MA, USA). DIFF responses were derived by subtracting the averaged condensation and rarefaction responses. For the continuous insertion monitoring recordings (Intraop1), averaged responses of two rarefaction and two condensation tonebursts with CRP, and 10 condensation and 10 rarefactions with AIM, at each insertion timestamp, were used. A Fast Fourier Transform (FFT) of the DIFF was computed with a rectangular window within a defined time range (CRP: 2 to 13 msec, AIM: 8 to 48 msec) to avoid onset/offset transients, and zero-padded to the next power of 2 before FFT. The amplitude and phase (relative to the acoustic stimulus) of each DIFF were obtained from the FFT bin closest to 500 Hz, hereafter referred to as “ECochG amplitude” and “ECochG phase.” Measurements were considered valid if the ECochG FFT amplitude was at least 1 μV (empirically determined), to ensure an ECochG potential above the noise floor. In addition, all recordings were visually inspected and approved. For each measurement, amplitude peaks across the different recording locations were identified based on the criteria defined by Bester et al. (2017). The number of valid peaks, the maximum amplitude, and the corresponding intracochlear electrode/timestamp were stored and the tonotopic frequencies of these electrodes/timestamps were derived from imaging data. Depending on the number of peaks, the recordings were grouped into the following patterns: flat peak pattern (no peaks), one peak pattern (1 peak), and multiple peak pattern (more than one peak). Recordings with at least one peak were further categorized based on the tonotopic frequency of the highest peak, into apical (<2000 Hz), mid (2000 to 6000 Hz), or basal (>6000 Hz) peak pattern. At last, surgical insertion approaches were categorized as cochleostomy versus RW insertion, with extended RW insertion grouped with RW insertion, as it is considered less traumatic than cochleostomy (Wang et al. 2017). EAs were classified by intracochlear position: perimodiolar (Contour Advance CI612), lateral wall (HiFocus SlimJ and Slim Straight CI622), and mid-scala (HiFocus MidScala).

### Statistical Analysis

Statistical analyses were conducted using R (version 4.4.1) and RStudio (version 2024.09.9), with a significance set at *p* < 0.05. Changes in maximum amplitude were analyzed with complete cases up to Postop1 using listwise deletion. Paired Wilcoxon signed-rank tests with Benjamini–Hochberg correction were used to compare Intraop1 versus Intraop2 and Intraop2 versus Postop1 amplitudes. A >30% amplitude reduction (based on the systematic review, Cooper et al. 2025) and >1 octave peak location shifts were treated as meaningful changes. A previous study examining peak-location shifts classified a shift as occurring when an apical peak, defined at electrode 20 or 22 (for CI422 or CI522 arrays), moved to a more basal peak at electrode 18 or below, meaning that a shift could be identified based on as little as a 2-electrode difference (Bester et al. 2023). In the current study, to also capture shifts in participants who already presented with a more basal peak pattern at baseline, a frequency-based criterion was used. To avoid classifying small fluctuations (e.g., those caused by postoperative electrode movements) as true peak shifts, a minimum shift of two recording electrodes was defined. For the CI622 electrode, which has the greatest inter-recording spacing among the arrays used, two recording electrodes correspond to approximately 3.6 mm along the BM, representing ~0.85 of an octave. This value was rounded to one octave (≈4 mm shift) and used as threshold. Post-hoc analysis compared peak tonotopic location (in octaves relative to the most apical electrode) between surgical insertion approaches (cochleostomy versus RW) using the Mann–Whitney *U* test, and across EA types (lateral wall, mid-scala, and perimodiolar) using the Kruskal–Wallis test. Significant Kruskal–Wallis test was followed by pairwise Mann–Whitney *U* tests with Benjamini–Hochberg multiple comparison correction. Effect sizes for Mann–Whitney *U* were expressed as the probabilistic index (*θ*), which quantifies the probability that a randomly selected value from one group will be greater than a randomly selected value from another group. *θ* was reported with its 95% confidence interval (95% CI), calculated using the DeLong method (DeLong et al. 1988) to account for imbalanced sample sizes (Perme & Manevski 2019). HP_1_ was compared between groups defined by the presence or absence of a >30% amplitude drop between consecutive recordings, and across peak location shift categories (apical, >1 octave shift in the apical direction; basal, >1 octave shift in the basal direction, or stable, <1 octave shift). Group differences were assessed with the Mann–Whitney *U* test (two groups) or the Kruskal–Wallis test (three groups, requiring ≥2 observations per group). The relationship between tonotopic peak location during Intraop1 and HP_1_ was tested using linear regression. All *p* values in analyses involving HP_1_ were corrected for multiple comparisons (Benjamini–Hochberg) and effect sizes (*θ*) with 95% CI were reported for Mann–Whitney *U* tests. Due to the small subgroup size at Postop2, no statistical analyses were performed for this timepoint.

## RESULTS

### Participant Information

A total of 80 participants were recruited for the study. Seven participants were excluded from all analyses due to intraoperative recording issues, that is, electrical interference artifacts during measurements (2 cases), intermittent contact to the implant (4 cases), or ear foam displacement (1 case). Intraoperatively, full insertion of the EA was achieved in all ears. Postoperative imaging revealed extracochlear electrodes in 3 cases and a tip fold-over in 1 case; these cases were additionally excluded from all analyses resulting in N = 69 participants. Table 1 summarizes the demographic data of these remaining 69 participants. Electrode dislocation into the scala vestibuli was detected in postoperative CT imaging in 4 participants. These participants were not excluded but visually indicated in the analyses. Supplemental Figure 1 in Supplemental Digital Content, https://links.lww.com/EANDH/B842, provides an overview of the included participants and the number of valid ECochG recordings. Intraoperatively, 10 Intraop1 recordings were excluded either due to loss of contact between the external and internal CI coils or electrical interference from artifacts affecting the ECochG potentials. During Intraop1, 83% of the participants (49/59) showed a valid ECochG potential. Intraop2 was conducted in all 69 participants, with 84% (58/69) of the recordings showing an ECochG amplitude above threshold (1 µV). All 58 participants with valid ECochG amplitude during Intraop2 were remeasured postoperatively (Postop1), with 55% (32/58) showing a valid ECochG amplitude at this recording timepoint. Postop2 was not performed in 13 participants due to participation in another study or loss to follow-up. Eighty-nine percent (17/19) of the participants with valid ECochG responses during Postop1 showed valid ECochG responses during Postop2.

**TABLE 1.** Overview of demographic data, side of implantation, EA type, surgical insertion approach, preoperative pure-tone average (0.125–8 kHz), low-frequency pure-tone average (0.25–1 kHz), cochlear duct length, insertion angle and tonotopic frequency of the most apical electrode, and the number of days between surgery and the postoperative measurements

	Overall (N = 69)
Age (yrs)	
Mean (SD)	62.3 (13.2)
Sex	
Male	33 (47.8%)
Female	36 (52.2%)
Etiology of hearing loss	
Idiopatic	52 (75.4%)
Infectious	3 (4.3%)
Menière disease	4 (5.8%)
Otosclerosis	2 (2.9%)
Sudden	6 (8.7%)
Trauma	2 (2.9%)
Side	
Left	33 (47.8%)
Right	36 (52.2%)
Electrode array	
CI 612	18 (26.1%)
CI 622	27 (39.1%)
MidScala	10 (14.5%)
SlimJ	14 (20.3%)
Surgical insertion approach	
Cochleostomy	15 (21.7%)
Extended RW	5 (7.2%)
RW	49 (71.0%)
Preoperative PTA (dB HL)	
Mean (SD)	85.6 (10.2)
Preoperative low-frequency PTA (dB HL)	
Mean (SD)	77.9 (14.7)
CDL_OC_ (mm)	
Mean (SD)	35.0 (1.68)
Insertion angle (°)	
Mean (SD)	406 (54.5)
Tonotopic frequency (Hz)	
Mean (SD)	816 (361)
Postop1 (days after surgery)	
Mean (SD)	51.9 (19.6)
Postop2 (days after surgery)	
Mean (SD)	353 (148)

CDL_OC_, cochlear duct length at the organ of Corti; CI, cochlear implant; EA, electrode array; Postop1, measurement 3 (postoperative sweep after approximately 7 wk); Postop2, measurement 4 (postoperative sweep after approximately 1 yr); PTA, pure-tone average; RW, round window.

The first postoperative audiogram showed that 7% of the participants had complete HP_1_ (>75%), 48% had partial HP_1_ (>25 to 75%), 10% had minimal HP_1_ (0 to 25%), and 35% had no measurable hearing at the maximum output of the audiometer. The mean HP_1_ was 29.5%, with a mean LF reduction of 20.0 dB, and a mean overall PTA reduction of 13.3 dB. In the subgroup of 19 participants who underwent the 1 yr assessment, 1 participant did not complete postoperative audiometric testing at that timepoint. Between the two postoperative timepoints, LF thresholds increased by a mean of 3.2, and the overall PTA increased by 2.8 dB. All performed audiograms are visualized in Supplemental Figure 2 in Supplemental Digital Content, https://links.lww.com/EANDH/B842.

### Example ECochG Recordings

**Fig. 1.**
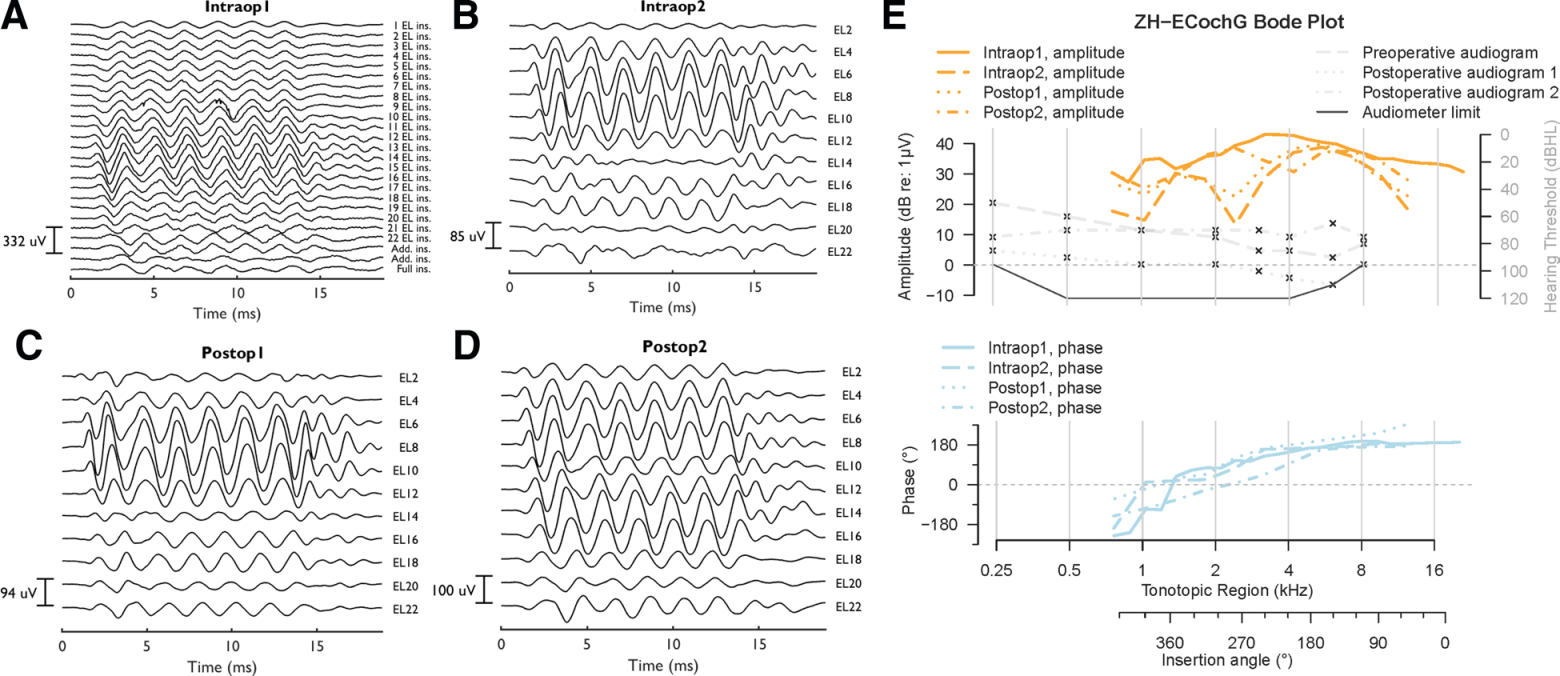
ECochG recordings from 1 participant at the different recording timepoints. A–D, DIFF curves extracted from the raw intracochlear ECochG recordings. Waveforms represent recording along the cochlear length, arranged from the most basal (top) to the most apical (bottom) recording location. A, The intraoperative Intraop1 recording at different timestamps during continuous insertion. B–D, The recordings obtained during Intraop2, Postop1, and Postop2 from the different intracochlear electrode contacts. Note the differences in scale for the different figures. E, Summary of the ECochG recordings using the ZH-ECochG Bode Plot that illustrates both the amplitude and the phase of the ECochG recordings (Geys et al. 2024). The top panel illustrates the pure-tone audiograms measured preoperatively, approximately 7 wk post-surgery, and about 1 yr post-surgery (shown in gray), with the audiometer limit indicated in black. In addition, the FFT amplitudes of the DIFF curves (dB relative to 1 µV) are plotted against insertion angles (degree) and the corresponding tonotopic frequencies (Hz) in orange. The bottom panel displays the phases (degree) of the DIFF curves against insertion angles (degree) and the corresponding tonotopic frequencies (Hz) for each recording timepoint in blue. Add. ins. indicates additional insertion; DIFF, difference curves; ECochG, electrocochleography; EA, electrode array; EL, electrode; FFT, Fast Fourier transform; Full ins., full insertion; Intraop1, measurement 1 (insertion monitoring); Intraop2, measurement 2 (intraoperative sweep after full insertion); Postop1, measurement 3 (postoperative sweep after approximately 7 wk); Postop2, measurement 4 (postoperative sweep after approximately 1 yr).

Figure 1 serves as an example of intracochlear recordings obtained from a single participant at the different ECochG recording timepoints. Figure 1A–D represents the waveforms (DIFF) of the ECochG responses for each recording timestamp or intracochlear electrode, and Figure 1E summarizes the ECochG recordings and pure-tone audiograms using the ZH-ECochG Bode plot (Geys et al. 2024). In this example, the Intraop1 amplitude data were classified as a mid-peak pattern, with 1 peak at 3176 Hz (angle of 241°) and a large spectral spread around this peak. During Intraop2, the peak pattern shifted slightly more basally. with the peak located at 5436 Hz (angle of 166°). The exact same pattern was observed during Postop1. In the final measurement (Postop2), the pattern changed to a 2-peak pattern, with an increase of magnitude at around 2416 Hz (angle of 278°) and a second peak at 7090 Hz (angle of 129°). The participant, diagnosed with Ménière disease, had a partial HP_1_ after surgery (36%) but showed improved hearing thresholds after 1 yr.

### Maximum Amplitude Changes

**Fig. 2.**
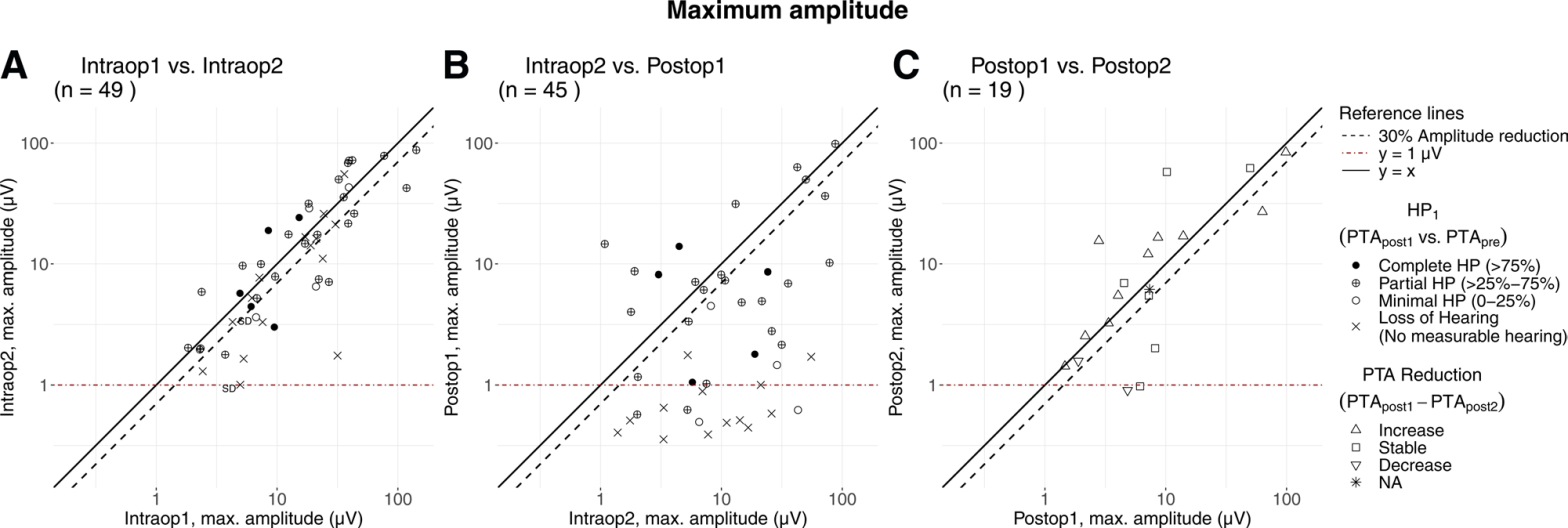
Scatter plots visualizing the change in maximum amplitude of the ECochG recordings between A, Intraop1 and Intraop2, B, Intraop2 and Postop1, and C, Postop1 and Postop2. Data points are only visualized if a valid ECochG potential was present during the measurement visualized on the *x* axis. Points are categorized based on HP categories, with (A–B) representing HP_1_ and (C) representing absolute PTA reduction between the two postoperative audiograms (increase ≥1 dB, decrease ≤1 dB, or stable). Instances of scalar dislocation are marked with SD. A diagonal line with a slope of 1 represents a stable maximum amplitude between 2 measurements, with the dashed line delineating a 30% reduction. Red dot-dashed line at *y* = 1 µV indicates the threshold of a valid ECochG potential. Note that the axes are displayed on a logarithmic scale. ECochG indicates electrocochleography; HP, hearing preservation; HP_1_, hearing preservation at the first postoperative measurement compared with the preoperative audiogram; Intraop1, measurement 1 (insertion monitoring); Intraop2, measurement 2 (intraoperative sweep after full insertion); max, maximum; Postop1, measurement 3 (postoperative sweep after approximately 7 wk); Postop2, measurement 4 (postoperative sweep after approximately 1 yr); PTA reduction, pure-tone average change between the first and the second postoperative audiogram; PTA_post1_, pure-tone average at Postop1 (0.125–8 kHz); PTA_post2_, pure-tone average at Postop2 (0.125–8 kHz); PTA_pre_, preoperative pure-tone average (0.125–8 kHz); SD, scalar dislocation.

Figure 2 illustrates changes in the maximum amplitude of ECochG recordings across different measurement stages using pairwise comparison. Figure 2A shows the comparison between Intraop1 (if valid) and Intraop2. Seventeen of the 49 participants (35%) exhibited a reduction in maximum amplitude of >30% during Intraop2 (below the dashed line), with 1 participant showing a complete loss of ECochG amplitude after Intraop1 (amplitude <1 µV, below red dot-dashed line). Notably, this participant also has a scalar dislocation of the EA, as observed in postoperative imaging (indicated as SD). The other participant with an identified scalar dislocation showed a >30% drop in maximum amplitude. The remaining 65% (32/49) of the data points remained above the 30% amplitude reduction boundary (dashed line), with 13 participants showing an increase of more than 30% between Intraop1 and Intraop2. Among the 13 participants with intraoperative amplitude increases, one showed a stable postoperative PTA (0.5 dB), with a low-frequency increase of 1.7 dB, whereas all others experienced postoperative PTA/LFPTA reduction. Participants who experienced complete postoperative hearing loss (HP_1_ = loss of hearing), 53% (8/15) remained above the 30% reduction threshold (above dashed line). Overall, no clear clustering by HP_1_ category was observed. Figure 2B compares the maximum amplitude between the Intraop2 (if valid) and Postop1. Participants for whom the acoustic sound pressure level could not be maintained during Postop1 were excluded for the visualization. A notable postoperative reduction in amplitude is observed, as 73% (33/45) showed a reduction of more than 30% (below the dashed line). A distinct cluster emerges as most participants who experienced a complete loss of ECochG responses (n = 19, amplitude <1 µV, below red dot-dashed line) also experienced a complete loss of hearing (12/19, HP_1_ = loss of hearing), whereas the remaining seven show some residual hearing (HP_1_ ≠ Loss of hearing). Seven participants showed a >30% increase in ECochG responses postoperatively compared with the Intraop2 measurements. Within this group, only 1 participant demonstrated an additional PTA improvement (2.5 dB), while the others had either a stable (n = 1) or reduced PTA (n = 5). In Figure 2C, 79% (15/19) of the participants showed no reduction greater than 30% between the first and second postoperative measurements (above the dashed line). Two participants completely lost ECochG responses at the second postoperative timepoint (amplitude <1 µV, below red dot-dashed line), of which one also had a decrease in PTA, and one had a stable PTA. One participant who experienced a PTA decrease after 1 yr retained stable ECochG signals over time (above the dashed line). At last, 6 participants showed a >30% increase in the ECochG amplitude between the postoperative measurements, 4 of whom also showed an increase in PTA. Overall, 6 of 19 participants showed a discrepancy between the change of amplitude and the change of PTA postoperatively.

**Fig. 3.**
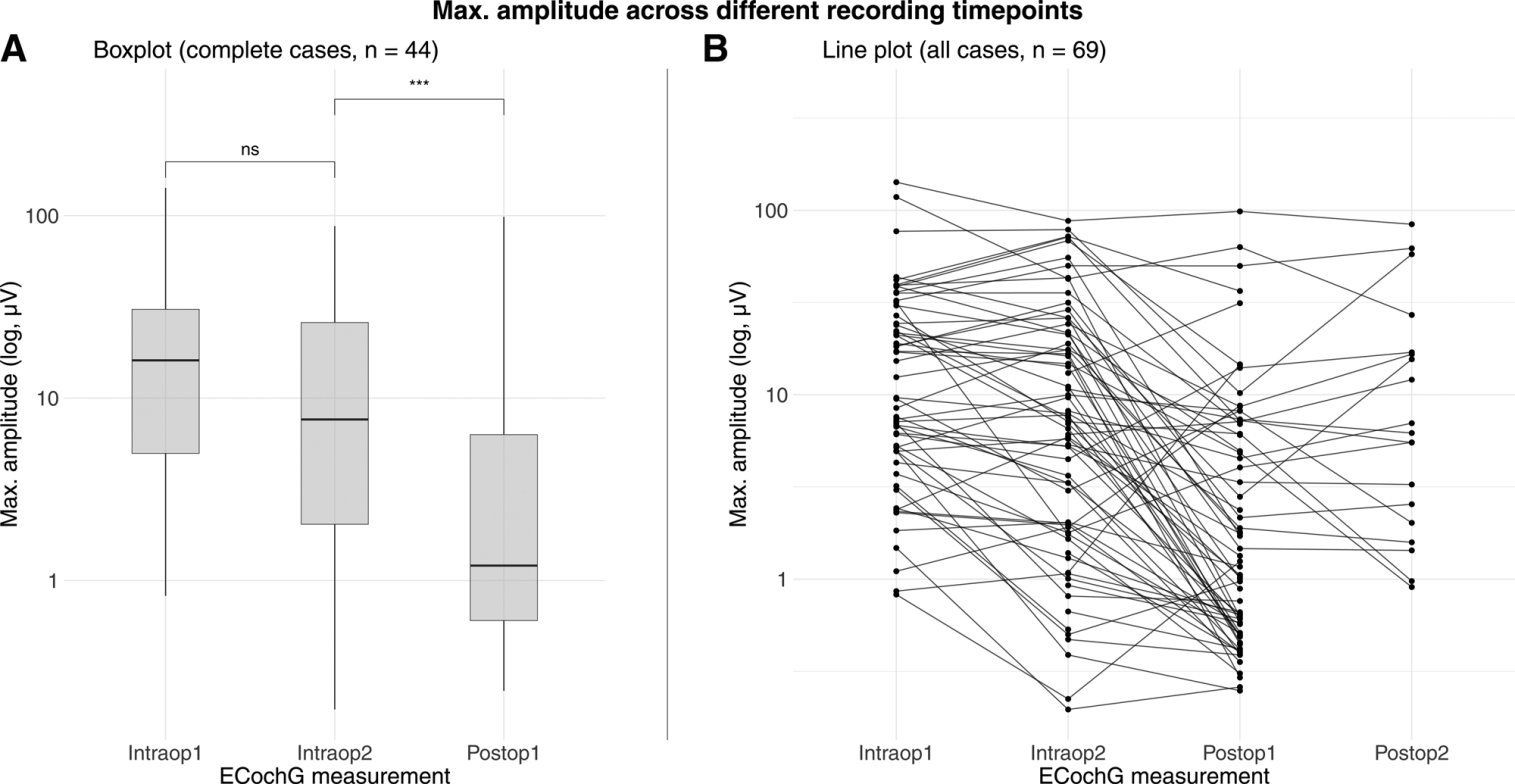
Maximum amplitude of the ECochG recordings across the different recording timepoints. A, Boxplot showing the maximum amplitude for the Intraop1, Intraop2, and Postop1 ECochG recording. The data represent only participants with complete data for all three timepoints and stable stimulus-level (complete cases analysis), independent of whether a valid ECochG potential was present or not. Statistical comparisons between the ECochG measurements were performed using a Wilcoxon signed-rank exact test (paired) and the significance levels are shown above the square brackets. *p* values were adjusted using the Benjamini–Hochberg method for multiple comparisons. The horizontal lines indicate the medians, the boxes indicate the 25th and 75th percentiles, and the whiskers indicate the range of nonoutlier data. B, Line plot showing the maximum amplitude across all 4 measurement timepoints, including all available data of each of the 69 participants (Postop1 is shown irrespective of invalid Intraop2 recordings or stimulus-level deviations). Each line represents an individual participant. Note that the maximum amplitudes are displayed on a logarithmic scale. ***Statistically significant (*p* < 0.001). ECochG indicates electrocochleography; Intraop1, measurement 1 (insertion monitoring); Intraop2, measurement 2 (intraoperative sweep after full insertion); max, maximum; ns, not significant; Postop1, measurement 3 (postoperative sweep after approximately 7 wk); Postop2, measurement 4 (postoperative sweep after approximately 1 yr).

Figure 3A presents a boxplot comparing maximum ECochG amplitude values across the first three measurements: Intraop1, Intraop2, and Postop1, including only participants with complete data for these three timepoints (n = 44), independent of whether a valid ECochG potential was present or not. The Wilcoxon signed-rank test (paired) showed no significant difference between ECochG amplitude during Intraop1 (median [interquartile range {IQR}] = 16.1 µV [25.8]) and Intraop2 (median [IQR] = 7.64 µV [24.0]) (*W* = 648, *p* = 0.075). However, a significant difference was observed between Intraop2 and Postop1 (median [IQR] = 1.21 µV [5.73]) (*W* = 826, *p* < 0.001). Figure 3B shows a line plot of the maximum amplitude across all four measurement timepoints, including all available data of each of the 69 participants. Each line represents an individual participant, and there is substantial interindividual variability in maximum ECochG amplitudes across timepoints. A strong downward shift is evident between the Intraop2 and Postop1 measurements, and different line trajectories are visible between Postop1 and Postop2.

### ECochG Amplitude Patterns

Various ECochG patterns were observed in this cohort. During Intraop1, an apical peak pattern was found in 59% (29/49) of the valid ECochG recordings with a median peak at 837 Hz. Mid-peak patterns were observed in 29% (14/49) with a median location of 3604 Hz, while basal peak patterns were seen in 12% (6/49) with a median tonotopic frequency of 7609 Hz. No recordings during Intraop1 showed a flat peak pattern. Mean preoperative thresholds were 83/71 dB HL (PTA/LFPTA) for the apical peak group, 89/88 dB HL for the mid peak group, and 84/82 dB HL for the basal peak group. Of the valid recordings during Intraop1, 27 (55%) showed a single peak, while 22 (45%) showed multiple peaks along the EA.

Supplemental Figure 3A in Supplemental Digital Content, https://links.lww.com/EANDH/B842, presents boxplots comparing the tonotopic location of the maximum peak amplitude relative to the tonotopic location of the most apical electrode (in octaves) across participants with a cochleostomy and RW insertion. The Mann–Whitney *U* test revealed no significant difference in relative peak location between cochleostomy (n = 9, median [IQR] = 1.67 octave [1.86]) and RW insertion (n = 40, median [IQR] = 0.94 octave [1.60]) (*W* = 209, *p* = 0.47, *θ* = 0.58 [95% CI: 0.37 to 0.79]). Supplemental Figure 3B in Supplemental Digital Content, https://links.lww.com/EANDH/B842, visualizes the difference in octaves between the tonotopic location of the peak and the tonotopic location of the most apical electrode for the three EA types. The Kruskal–Wallis test showed a significant difference between the groups (χ^2^ = 7.22, degrees of freedom = 2; *p* = 0.027). Post-hoc pairwise comparison revealed a significant difference in relative tonotopic ratio between EA types positioned at the lateral wall (n = 33, median [IQR] = 0.52 octave [1.67]) and those positioned in the middle of the scala (n = 8, median [IQR] = 1.83 octave [1.37]) (*W* = 58, *p* = 0.047, *θ* = 0.78 [95% CI: 0.62 to 0.94]). No significant differences were found between lateral wall and perimodiolar EA types (n = 8, median [IQR] = 1.33 octave [0.94]) (*W* = 89, *p* = 0.19, *θ* = 0.66 [95% CI: 0.47 to 0.85]), or between EA types positioned close to the modiolus and those in the middle of the scala (*W* = 45, *p* = 0.19, *θ* = 0.70 [95% CI: 0.42 to 0.99]).

### Maximum Amplitude Location Changes

**Fig. 4.**
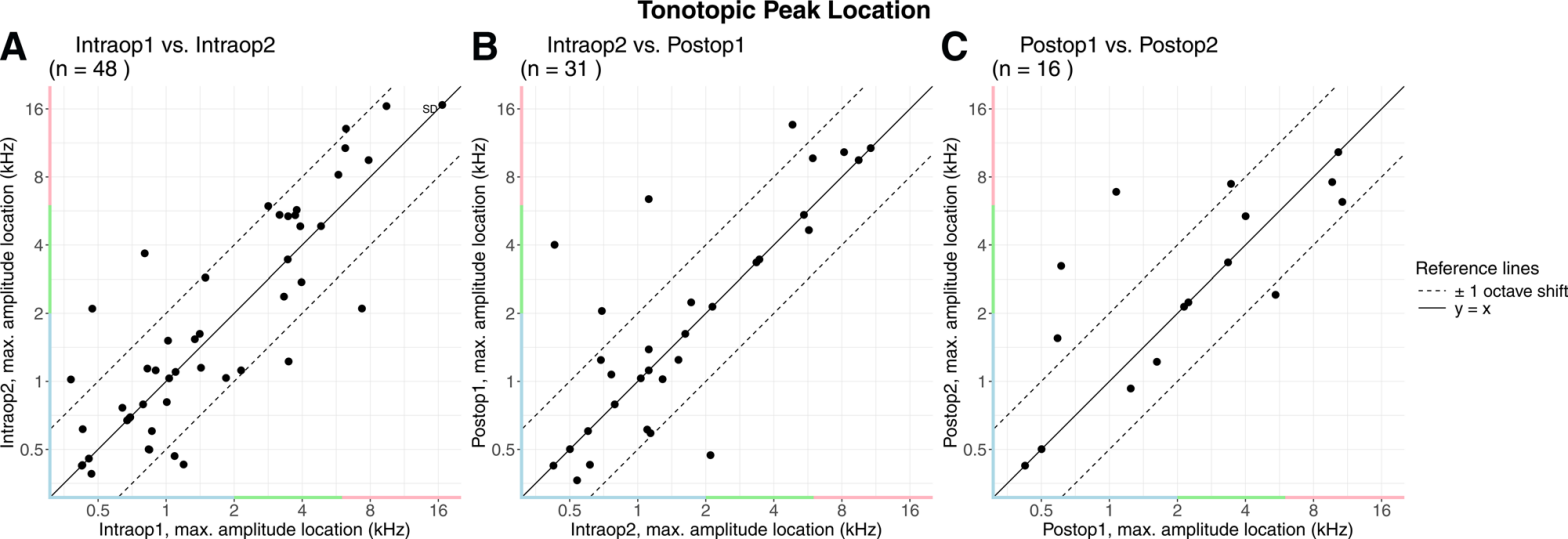
Scatter plots visualizing the shift in tonotopic location of the maximum amplitude of the ECochG recordings between A, Intraop1 and Intraop2, B, Intraop2 and Postop1, C, Postop1 and Postop2. Data points are only visualized if a valid amplitude peak could be defined along the cochlear length for both recordings. Instances of scalar dislocation are marked with SD. A diagonal line with a slope of 1 represents a stable maximum amplitude location between 2 measurements, with the dashed lines indicating a frequency shift of 1 octave. The blue, green, and red axes indicate an apical, mid, and basal peak pattern, respectively. ECochG indicates electrocochleography; Intraop1, measurement 1 (insertion monitoring); Intraop2, measurement 2 (intraoperative sweep after full insertion); max, maximum; Postop1, measurement 3 (postoperative sweep after approximately 7 wk); Postop2, measurement 4 (postoperative sweep after approximately 1 yr); SD, scalar dislocation.

The location of the maximum amplitude across different recording timepoints is visualized in Figure 4. Data points are only visualized if a valid amplitude peak could be defined at the different recording timepoints. Figure 4A compares the maximum amplitude location during Intraop1 with the Intraop2 (n = 48). The maximum amplitudes of the participants are distributed across the apical (blue), mid (green), and basal (pink) regions of the cochlea. A stable peak location is observed between Intraop1 and Intraop2, as indicated by the majority of the points (81%, 39/48) clustering around the diagonal within the 1-octave boundaries. The mean absolute log2 shift was 0.59 octaves (angle: 51°, distance: 2.66 mm). Nineteen percent of the participants (9/48) showed a shift of more than 1 octave of the maximum amplitude between Intraop1 and Intraop2 recording. Shifts were seen in both directions: basal shifts (5/48) and apical shifts (4/48). Figure 4B examines the relationship between the Intraop2 and Postop1 (n = 31). Here, the majority (84%, 26/31) showed a stable peak location of the maximum amplitude, with a mean absolute log2 shift of 0.57 octaves (angle: 47°, distance: 2.56 mm). Sixteen percent (5/31) showed a location shift >1 octave, with four participants showing a basal shift, and 1 participant an apical shift. Figure 4C compares Postop1 and Postop2 (n = 16). Sixty-nine percent (11/16) showed a stable peak location, with a mean absolute log2 shift of 0.70 octaves (angle: 61°, distance: 3.31 mm), while 4 participants showed a basal shift, and 1 participant showed an apical shift of the maximum amplitude.

### Residual Hearing

**Fig. 5.**
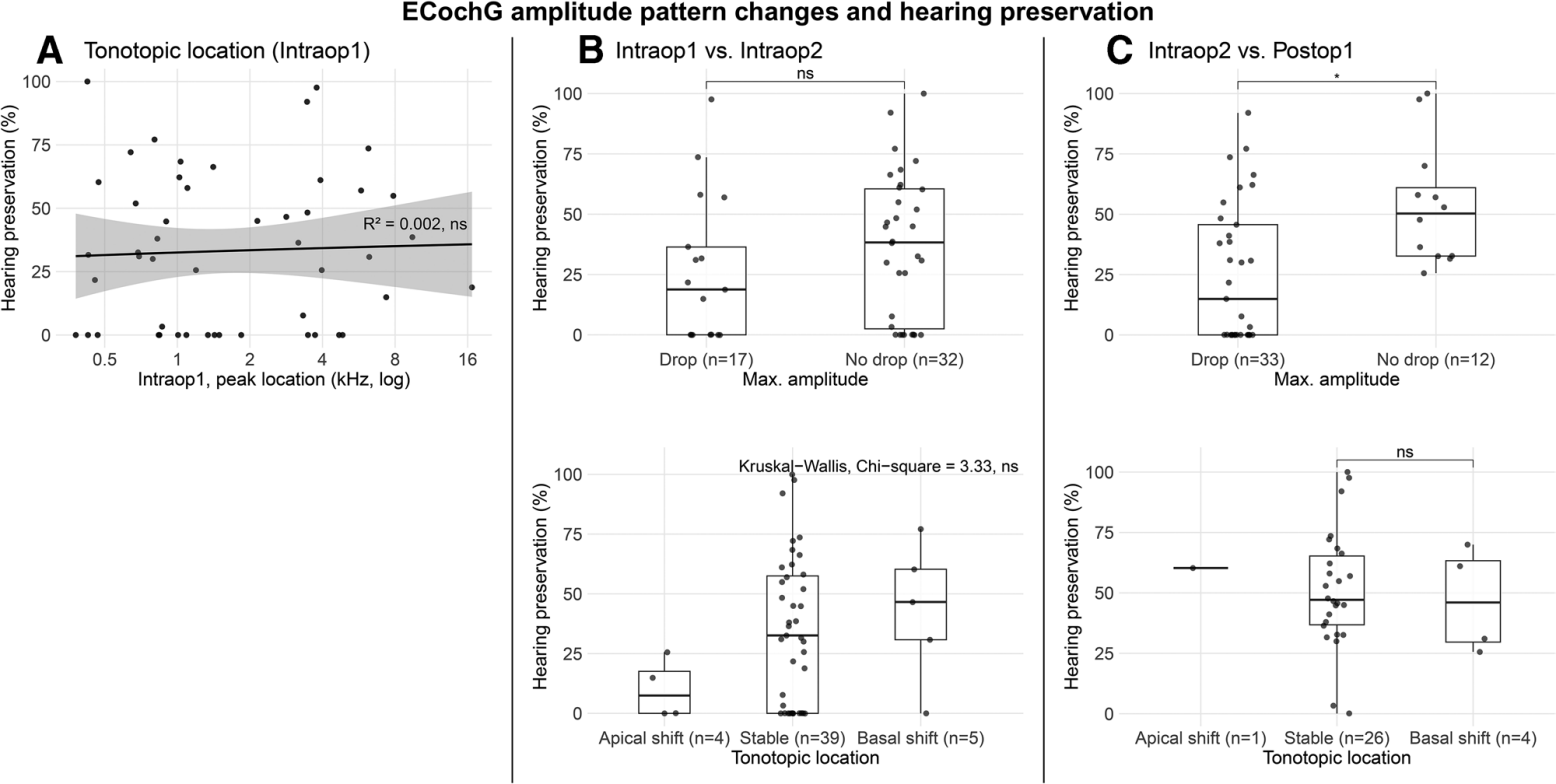
ECochG amplitude pattern changes and postoperative HP_1_. A (top left), Scatterplot showing the relationship between tonotopic peak location during Intraop1 (log-transformed) and postoperative HP_1_. B, (Top) Boxplot comparing HP_1_ between cases with and without a >30% drop in maximum amplitude between Intraop1 and Intraop2. (Bottom) Boxplot showing HP_1_ across three categories of tonotopic shift from Intraop1 to Intraop2: >1 octave apical shift, stable location, and >1 octave basal shift. C, (Top) Boxplot comparing HP_1_ between cases with and without a >30% drop in maximum amplitude between Intraop2 and Postop1. (Bottom) Boxplot comparing HP_1_ across tonotopic shift categories from Intraop2 to Postop1. Horizontal lines indicate medians, boxes represent interquartile ranges, and whiskers represent the range of nonoutlier data. Statistical tests included linear regression, Mann–Whitney *U* test, and Kruskal–Wallis test, with *p* values adjusted using the Benjamini–Hochberg method. *Statistically significant (*p* < 0.05). ECochG indicates electrocochleography; HP_1_, hearing preservation; Intraop1, measurement 1 (insertion monitoring); Intraop2, measurement 2 (intraoperative sweep after full insertion); max, maximum; ns, not significant; Postop1, measurement 3 (postoperative sweep after approximately 7 wk).

No association was found between the tonotopic peak location during Intraop1 and HP_1_ (*R*² = 0.002; *p* = 0.77) (Fig. 5A). HP_1_ did not differ between cases with (n = 17) versus without (n = 32) a >30% amplitude drop from Intraop1 to Intraop2 (*W* = 207, *p* = 0.32, *θ* = 0.62 [95% CI: 0.45 to 0.79]) (Fig. 5B, top). In contrast, cases with an amplitude drop (n = 33) from Intraop2 to Postop1 had significantly lower HP_1_ (median [IQR] 14.9% [45.7]) compared with those without a drop (n = 12) (median [IQR] 50.3% [28.3]) (*W* = 93, *p* = 0.028, *θ* = 0.77 [95% CI: 0.63 to 0.90]) (Fig. 5C, top). No significant differences in HP_1_ were observed across categories of tonotopic shift between Intraop1 and Intraop2 (χ² = 3.33, degrees of freedom = 2; *p* = 0.32) or between Intraop2 and Postop1 (*W* = 60, *p* = 0.77, *θ* = 0.58 [95% CI: 0.19 to 0.96]) (Fig. 5B–C, bottom).

## DISCUSSION

This longitudinal study is the first to monitor ECochG amplitude patterns along the cochlear duct, from CI electrode insertion through 1 yr postimplantation, by recording from multiple electrode locations rather than relying solely on recordings from a single recording electrode. CT-based tonotopic mapping of electrode positions was incorporated to account for variability in EA type, anatomical differences, and therefore individual electrode placement. While ECochG amplitudes remained largely stable intraoperatively, significant reductions were observed in the early postoperative phase, with some changes continuing up to 1 yr postimplantation. Furthermore, nonapical peak patterns were frequently observed in the cohort; however, no relationship was found between peak location and HP_1._ In a subset of cases, shifts in amplitude peak location were detected in both directions, even 1 yr after surgery. While these alterations in ECochG amplitude pattern, both maximum amplitude and tonotopic location, were hypothesized to reflect intracochlear changes, only the reduction in maximum amplitude between Intraop2 and Postop1 showed a significant association with HP_1_. Specifically, participants with a >30% decrease in maximum amplitude showed poorer HP_1_ measured around 7 wk postsurgery compared with those whose amplitude remained stable over the same period.

### ECochG Amplitude Patterns

An 83% success rate in obtaining ECochG potentials during EA insertion was found, slightly lower than the 91.8% average reported by Yin et al. (2021) in a systematic review including 12 studies performing intracochlear measurements during insertion. The slightly lower success rate can be attributed to the broad inclusion criteria for preoperative residual hearing in the present study (mean PTA of 85.6 dB HL and LFPTA of 77.9 dB HL), as it is known that higher ECochG amplitudes are typically obtained in individuals with better preoperative hearing (Andonie et al. 2025). Apical peak patterns were most common during insertion monitoring, though mid and basal peak patterns also occurred with notable frequency. This distribution is consistent with Harris et al. (2017a), despite differences in classification criteria, yet the high proportion of nonapical patterns is unexpected under low-frequency (500 Hz) stimulation. Even when accounting for the level-dependent basalward shift of the best-frequency location described by Walia et al. (2025) and Sagi and Svirsky (2025), the ~1.1 octave shift at high intensity (500 Hz) seen in ECochG data by Walia et al. would place the peak near 1084 Hz (Greenwood), which still falls within the apical category in our study. The apical peak pattern group showed slightly better LFPTA than the mid and basal groups, consistent with Harris et al., who reported poorer preoperative PTA (0.5 to 2 kHz) in nonapical peak patterns and with Dalbert et al. (2025) and Haumann et al. (2024a), who found parallels between ECochG patterns and audiometric thresholds. Tonotopic location of the peak during Intraop1 only explained 0.2% of the variance in HP_1_. Studies by Buechner et al. (2022), Giardina et al. (2019), and Walia et al. (2022) likewise reported that ECochG amplitude patterns were insufficient for detecting insertion damage. In contrast, Bester et al. (2023) reported greater hearing loss in participants showing a more basal peak pattern in intraoperative postinsertion sweep recordings, and Andonie et al. (2025) found that participants with a peak located further from the expected tonotopic location of the acoustic stimulus tended to have poorer postoperative residual hearing. Our study results suggest that such patterns are not necessarily markers of insertion trauma and may instead reflect variability in cochlear integrity (e.g., dead regions). Peak pattern classifications were based on the highest peak, although in 45% of the recordings during Intraop1, more than one amplitude peak along the cochlear duct was found. Such additional peaks may arise from using high stimulation levels, potentially activating more basally located hair cells and creating destructive interference patterns due to phase difference (Giardina et al. 2019) or due to cross-turn sensitivity influenced by the specific cochlear geometry (van Gendt et al. 2020). Confirming interference effects through phase analysis, however, was beyond the scope of this study.

Post-hoc analyses in this study showed that the surgical insertion approach did not influence the peak location in the cohort, although the wide 95% CI for *θ* [0.37 to 0.79] suggests uncertainty around the true effect. These results are consistent with Ramos-Macias et al. (2019), who evaluated 15 CI participants. In contrast, EA type influenced the peak location, with mid-scala EAs tending to show a more basal peak compared with lateral wall arrays. The 95% CI of the effect size (95% CI: 0.62 to 0.94) indicates a moderate separation despite the small sample size of 8 mid-scala EAs. Other comparisons did not reveal significant differences in maximum peak locations, although the median peak location was more basal for perimodiolar EAs compared with lateral wall EAs. Larger cohorts will be needed to confirm these effects. Giardina et al. (2019) observed response drops during insertion after stylet removal with perimodiolar EAs, which could be seen as a nonapical peak. Similarly, in the current study, a stylet was removed during insertion for precurved EAs (Contour Advance CI612 and HiFocus MidScala EA). It is known that, if the precurved EA contacts the outer wall of the first cochlear turn before stylet removal, this could result in contact, or even perforation, between the EA and the BM and/or osseous spiral lamina (Rebscher et al. 2008). The curvature of the first cochlear turn begins at approximately 180° of insertion depth (Dhanasingh & Jolly 2017), potentially explaining more basally located peaks. Radiological examination in the current cohort revealed EA dislocation into the scala vestibuli in 4 participants; a more traumatic disruption of the BM movement that could explain a reduction in ECochG amplitude apical to the translocation (Koka et al. 2018). While translocation may account for the nonapical peak pattern observed in the 2 cases with valid intraoperative ECochG responses, it does not explain the high prevalence of this pattern in the cohort.

### ECochG Changes Immediately Following EA Insertion

Maximum amplitude remained stable on average after EA insertion, although 35% showed a >30% intraoperative reduction, including 2 cases with scalar dislocation, suggestive of acute trauma. However, no significant difference in postoperative HP_1_ could be found between participants with and without an intraoperative maximum amplitude drop. This intraoperative stability in ECochG amplitude in the majority of the cases could be interpreted as low-impact EA insertion and aligns with findings by Riggs et al. (2019), who compared the amplitude at a basal location in the cochlea, as well as Saoji et al. (2023), who compared the peak amplitudes between the final ECochG recording during insertion and the apical recording from the sweep measurement, reporting only 1 participant (of 10) with a decrease in ECochG peak amplitude between the timepoints. The absence of difference in HP_1_ between the participants with and without an intraoperative amplitude drop is consistent with Riggs et al., who reported no relationship between changes in ECochG amplitude across the two intraoperative recording timepoints and the percentage of low-frequency hearing loss. In some cases, an increase in maximum amplitude was observed between the intraoperative recordings, but only in 1 participant was this increase linked with improved postoperative residual hearing. This increase could hypothetically reflect a dissolution of air bubbles within the cochlea or the release of a BM fixation that allowed movement to resume. Continuous impedance recordings are needed to validate this hypothesis.

Similar to the maximum amplitude findings, the tonotopic location of the maximum amplitude was generally stable (in 81%) after EA insertion, which is consistent with Soulby et al. (2021), who reported a significant correlation between the maximum amplitude location during intraoperative insertion and postinsertion sweep recordings, with a 29° mean absolute error in their cohort of 14 ears. In the current cohort, the mean absolute shift was higher (51° or 2.66 mm), possibly due to the larger sample size and the inclusion of different EAs with varying recording contact spacings. In the 9 participants with a shift greater than one octave, both apical and basal location shifts were observed. These shifts may be due to EA fixation during insertion, which could resolve upon full insertion or vice versa, as the EA is still manipulated by the surgeon between the two intraoperative recordings to secure the hold of the array. No significant difference in HP_1_ was found between the three peak location shift groups, indicating that a significant shift of peak location intraoperatively is not a sufficient marker for postoperative reduction of residual hearing, although larger group sizes with peak changes are needed to draw final conclusions.

### Early Postoperative ECochG Changes

Although on average maximum amplitude remained stable intraoperatively, a significant early postoperative reduction was observed in the Postop1 amplitude (*p* < 0.001). This pattern, consistent with previous studies (Dalbert et al. 2015; O’Connell et al. 2017; Ramos-Macias et al. 2019; Valenzuela et al. 2021), likely reflects early inflammatory responses, intracochlear foreign body reactions, or neural and/or sensory cell degeneration (Kel et al. 2013; O’Leary et al. 2013; Kopelovich et al. 2015; Rahman et al. 2022). In addition, participants with a drop of >30% also showed poorer HP_1_ (*p* = 0.028). This relationship is physiologically plausible, as a marked amplitude loss in the early postoperative phase is likely to correspond to compromised cochlear function, detectable with behavioral thresholds. Previous studies have demonstrated similar findings, showing correlations between changes in ECochG at the most apical electrode and corresponding changes in behavioral thresholds (Abbas et al. 2017; Tejani et al. 2021). Within the cohort, 45% of all study participants with valid intraoperative ECochG recordings (Intraop2) lost their ECochG signals completely within the first weeks after surgery (Postop1). In the prospective study by Bester et al. (2023), sweep measurements were taken intraoperatively, immediately after implantation, and repeated 3 mo postimplantation. In their study, interpretable ECochG responses were still present in all 39 participants after 3 mo. This difference may be explained by the more homogenous cohort studied by Bester et al., who included only participants with preoperative hearing thresholds ≤80 dB HL at 0.5 kHz and receiving lateral wall electrodes; a much stricter criterion than in the present study. In the current cohort, preoperative PTA and LFPTA were poorer in the group that lost the ECochG signals at Postop1 (nonvalid) and the group that still had valid ECochG potentials (valid) (PTA: 87.0 versus 81.4 dB HL and LFPTA: 79.7 versus 71.6 dB HL). These findings suggest that the higher rate of ECochG loss may be due to the inclusion of participants with minimal residual hearing, who inherently have less cochlear function to lose. Despite the large number of participants showing a reduction of amplitude in the early postoperative stage, some participants showed an increase in maximum amplitude, although only one also showed an improvement in residual hearing. ECochG increase could indicate intracochlear recovery, but this interpretation remains tentative without supporting measurements such as impedance recordings.

In most participants (84%), the ECochG peak location remained stable between intraoperative and early postoperative measurements. Shifts >1 octave generally reflected a more basal peak, possibly due to intracochlear changes such as fibrosis that affect travelling wave propagation to the apex. Consistent with this, Bester et al. (2023) associated apical-to-mid peak shifts (and stable mid peaks) with higher four-point impedances, suggestive of increased fibrosis. In the current cohort, however, peak shifts did not correspond to poorer HP_1_. This contrasts with Bester et al., who linked basal shifts to greater hearing loss at 3 mo. The small number of participants with peak shifts in the present study contributed to wide 95% CIs around the effect size and may explain the absence of an observed association.

### Late Postoperative ECochG Changes

Although 1-yr follow-up ECochG recordings were available only in a small subset (n = 19), maximum amplitudes showed different trajectories, with some increasing, others decreasing, and several remaining stable over the postoperative time period. Overall, the median maximum amplitude remained relatively unchanged (decrease of 0.9 µV). Valenzuela et al. (2021) reported a decrease of the median ECochG total response amplitudes (n = 20) over time, with the final measurement taken after 12 mo, although their analysis was limited to the apical electrode, providing no information on potentially larger responses from more basal sites. In most of the participants, an agreement between amplitude changes and PTA changes was detected (e.g., a decrease of maximum amplitude with a decrease of PTA). This is similar to the findings of Tejani et al. (2023), who found stable amplitudes recorded from the most apical electrode in participants with stable residual hearing, and a reduction in amplitude in the group with delayed hearing loss. Due to the limited number of valid Postop1 recordings, the relationship between postoperative hearing and ECochG amplitude changes over time was not statistically analyzed in the current study. By 1 yr, most participants showed stable peak locations, although 5 of 16 experienced a peak shift. Changes in both maximum amplitude and peak location still occurred in the postoperative phase, suggesting that intracochlear changes continue beyond the early postoperative period. These may reflect either resolution of inflammation or intracochlear tissue growth.

### Limitations

While this study provides valuable insights into ECochG amplitude patterns, several limitations should be acknowledged. The analysis was restricted to the magnitude of the DIFF response using a single low-frequency, high-intensity stimulus; additional parameters (e.g., phase, neural contributions) and varying frequencies or intensities could yield further insights. The estimated tonotopic location could be imprecise as all ECochG recordings per participant were linked to a single postoperative CT scan, which does not account for potential EA movement over time. In addition, intraoperative monitoring involved fewer averages, a moving EA, and a delay between the surgeon’s verbal electrode identification and the ECochG timestamps, likely introducing variability of the precise location and noisier recordings. Robot-assisted insertion could help mitigate this issue by enabling slower, more controlled advancement. The broad inclusion criteria of this study (etiology, preoperative hearing levels, and EA types) strengthened generalizability but added complexity to interpretation. Moreover, the use of different manufacturer-specific ECochG systems limited full alignment of recording parameters, though this likely had minimal impact as analyses were primarily conducted within participants. An additional consideration is postoperative middle ear effusion, which is expected to be minimal or absent at 7 wk postoperative based on findings by Dalbert et al. (2015), but could still have influenced the comparisons, as air–bone gaps could not be checked in most participants due to bone conduction output limits. Future studies should include tympanometry or otoscopy at follow-up to rule out this factor. Finally, due to the longitudinal design of this study, data exclusions were necessary for various reasons, including participant dropouts, invalid or lost ECochG recordings, and variations in sound pressure levels across consecutive measurements. As a result, the power of some analyses was reduced.

### Implications and Future Directions

A variety of ECochG amplitude patterns was observed, with nonapical peak patterns being relatively common. The absence of association between the location of the peak and postoperative HP_1_ leaves uncertainty about what the amplitude patterns reflect, suggesting that they are not necessarily markers of insertion trauma and may instead reflect variability in cochlear integrity. As such, the use of ECochG traces during EA insertion or as a local measure of cochlear integrity warrants further investigation. Within the cohort, most ECochG amplitude patterns changed in the early postoperative stages, which was associated with worse HP, despite showing stable amplitude patterns intraoperatively. These findings support further investigation into interventions to minimize early intracochlear processes, such as drug-eluting EAs or intracochlear catheters (Prenzler et al. 2018; Parys et al. 2022; Fleet et al. 2024). In addition, changes in both the magnitude of the responses and the location of the peak were observed when comparing different ECochG recordings, suggesting ongoing intracochlear alterations, even in the late postoperative stage. Although the current study used a threshold of one octave to define a meaningful peak-location shift, the use of this criterion remains under debate and warrants further investigation. Overall, the study highlights the value of ECochG in monitoring intracochlear processes, although correlations with other biomarkers of intracochlear changes (e.g., four-point impedance measurements) should be investigated to fully validate its utility. Finally, using the tonotopic location along the EA enabled a comparison of different EA types from various manufacturers, providing insight into ECochG amplitude patterns relative to the acoustic stimulus’ tonotopic location. Applying this method in future studies could facilitate cross-study comparisons and further improve the understanding of these amplitude patterns.

## ACKNOWLEDGMENTS

The authors gratefully acknowledge the cochlear implant users who participated in this study, as well as the clinical team of the cochlear implant center and the hospital for their great support during the study.

## Supplementary Material
