# Supplementary material for: Longitudinal Analysis of Intracochlear Electrocochleographic Amplitude Patterns in Cochlear Implant Recipients
Source: Ear Hear. 2026 Feb 6;47(4):1003–15. doi: 10.1097/AUD.0000000000001793 (PMC13252957; doi:10.1097/AUD.0000000000001793)
Supplement: Supplementary file 1 [file aud-47-1003-s001.pdf]

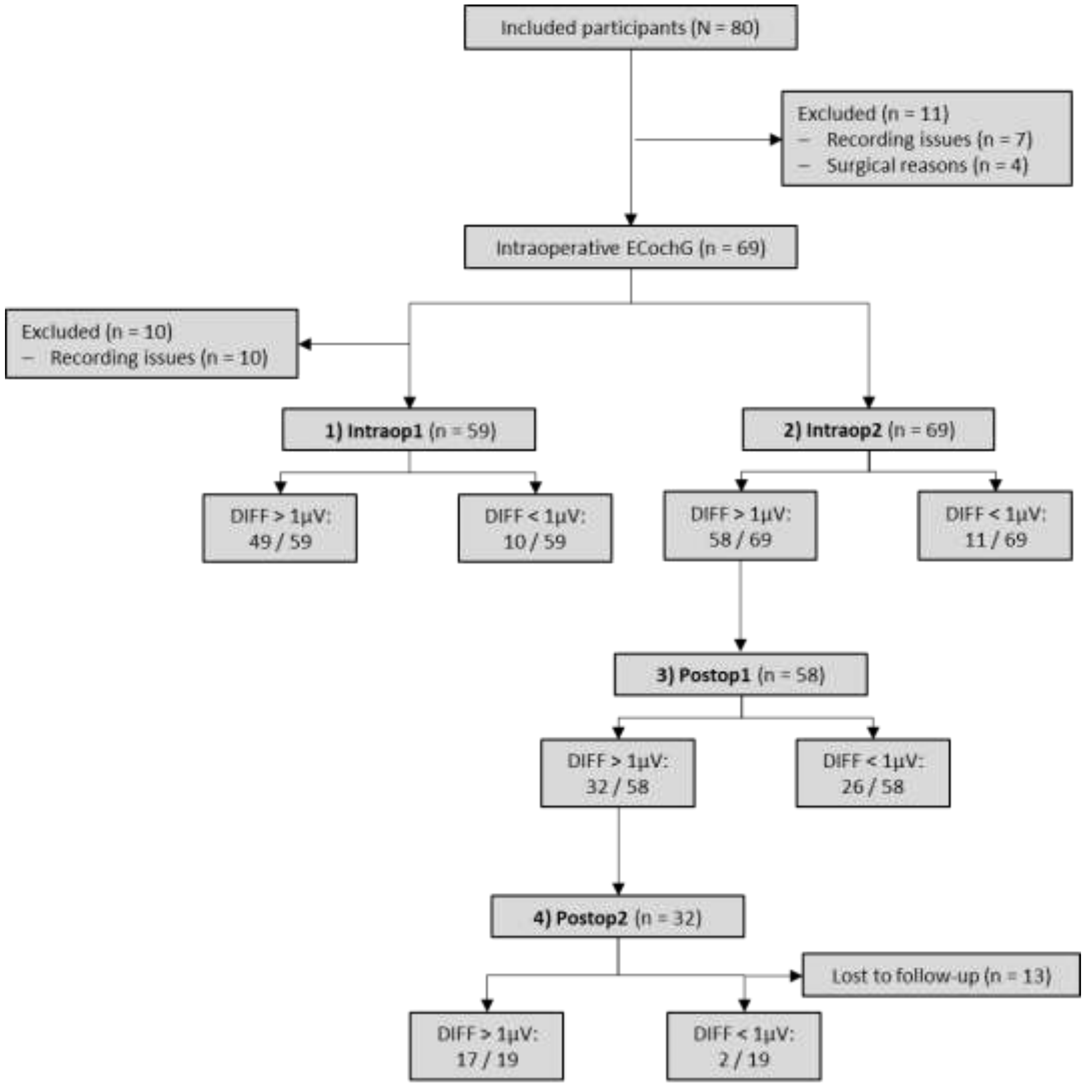

Supplemental Figure 1. Overview of the ECochG recordings available for analysis and the number of valid ECochG potentials. A valid recording is defined as having an FFT amplitude of the DIFF that is at least 1 µV, combined with a successful visual inspection of the waveforms. Only participants with a valid ECochG recording were included in the subsequent measurement. DIFF indicates difference curve; ECochG, electrocochleography; FFT, fast fourier transform; Intraop1, measurement 1 (insertion monitoring); Intraop2, measurement 2 (intraoperative sweep after full-insertion); Postop1, measurement 3 (postoperative sweep after approximately 7 wk); Postop2, measurement 4 (postoperative sweep after approximately 1 yr)

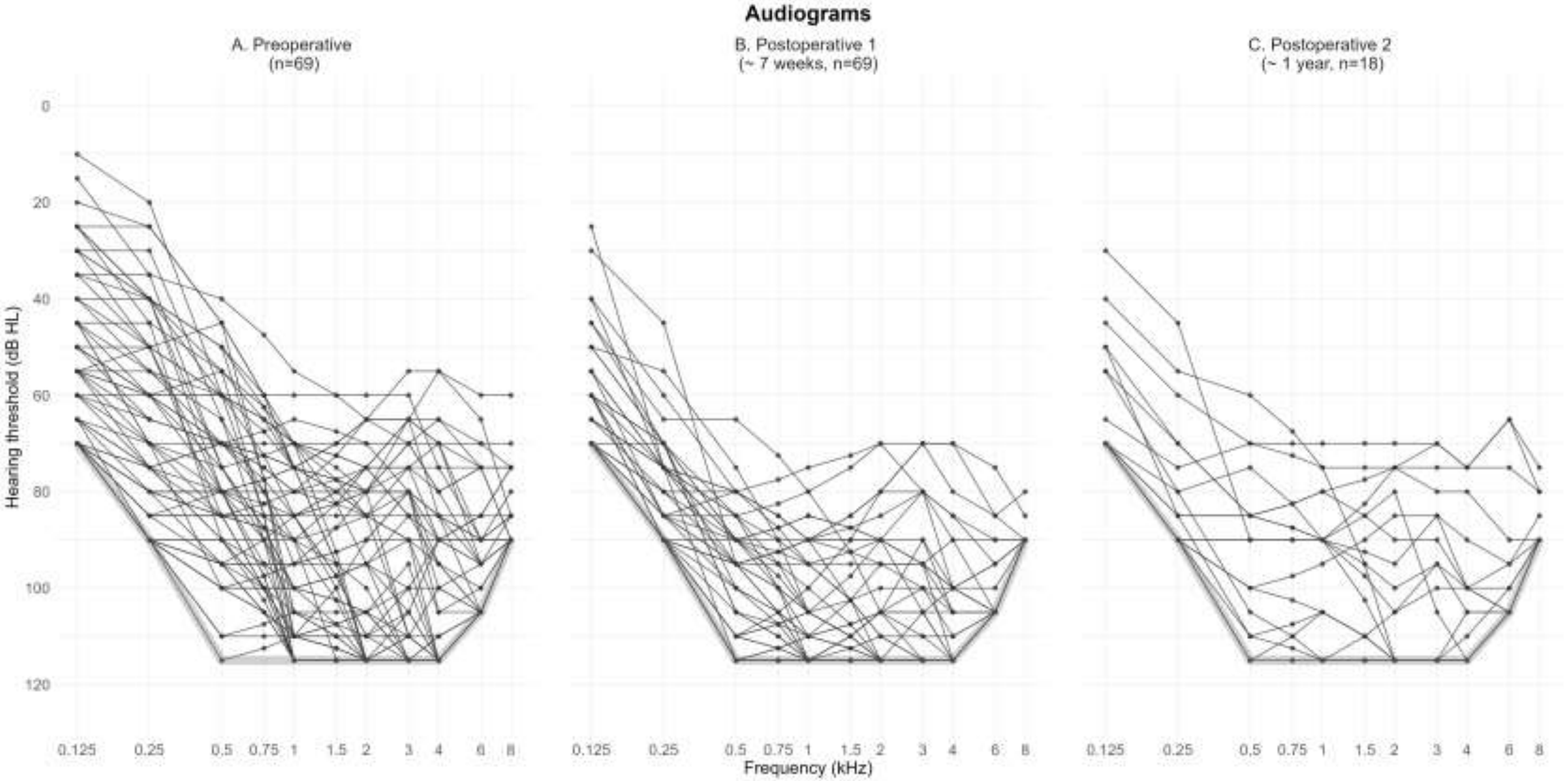

Supplemental Figure 2. Line plots showing the air-conduction thresholds of all included participants. A, preoperatively, B, early postoperatively (approximately 7 wk after surgery), C, and late postoperatively (approximately 1 yr after surgery). Postoperative audiograms at the one-year timepoint were only collected for participants who underwent ECochG recordings at that time.
dB HL indicates decibel hearing level; kHz, kilohertz.

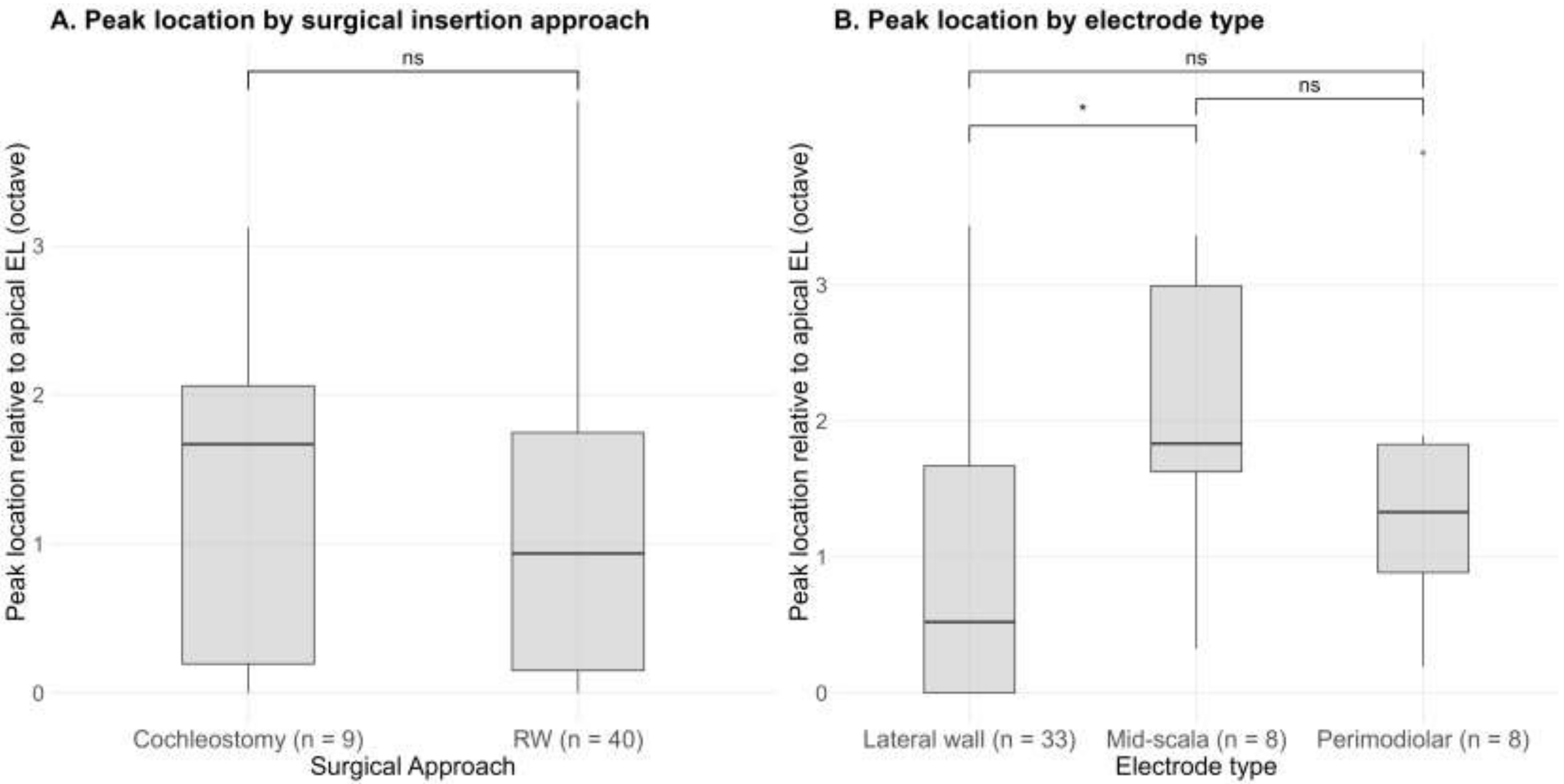

Supplemental Figure 3. Boxplot showing the relative ratio (in octaves) of the peak tonotopic location during insertion monitoring (Intraop1) relative to the tonotopic location of the apical electrode, for A (left), two surgical insertion approaches (cochleostomy and RW) and for B (right), the three EA types: lateral wall, mid-scala, and perimodiolar. The horizontal lines indicate the medians, the boxes indicate the 25th and 75th percentiles, the whiskers indicate the range of non-outlier data, and the dots indicate outliers. Significance levels of the used Mann-Whitney U test (left, A) and of the pairwise comparison with post-hoc multiple testing correction (Benjamini-Hochberg) (right, B) are shown above the square brackets.

*Statistically significant ($p < 0.05$). EA indicates electrode array; EL, electrode; RW, round window; Intraop1, measurement 1 (insertion monitoring); ns, not significant.
